# Investigation of Two Novel Heterojunction Photocatalysts with Boosted Hydrogen Evolution Performance

**DOI:** 10.3390/nano14231947

**Published:** 2024-12-04

**Authors:** Kaifeng Zhang, Xudong Wang, Yanjing Su

**Affiliations:** 1 Beijing Advanced Innovation Center for Materials Genome Engineering, Institute for Advanced Materials and Technology, University of Science and Technology Beijing, Beijing 100083, China; zhangkf2017@163.com; 2 Corrosion and Protection Center, University of Science and Technology Beijing, Beijing 100083, China; xdwang@ustb.edu.cn

**Keywords:** composite materials, semiconductors, nanocomposites, photocatalysis

## Abstract

Among the reported photocatalysts, ZnIn_2_S_4_ has garnered significant research interest due to its advantageous layered structure and appropriate band gap. However, achieving rational design and effective interfacial regulation in heterojunctions remains challenging. In this study, we designed two novel heterojunctions: SrTiO_3_@ZnIn_2_S_4_ and SrCrO_3_@ZnIn_2_S_4_. The photocatalytic hydrogen evolution performance of prepared heterojunctions was systematically investigated under different single-wavelength light sources. Without a cocatalyst, the optimized hydrogen evolution efficiency of SrTiO_3_@ZnIn_2_S_4_ and SrCrO_3_@ZnIn_2_S_4_ reached 3.27 and 4.6 mmol g^−1^. The enhanced photocatalytic performance can be attributed to the formation of a type-II heterojunction, which improves light absorption capabilities and promotes the separation and transfer of photoinduced carriers. This study provides valuable insights into the strategic construction of heterojunctions for photocatalytic water splitting.

## 1. Introduction

In the context of carbon neutrality, hydrogen is regarded as a sustainable clean energy to alleviate environmental pollution resulting from fossil fuel consumption [1,2,3]. Powered by solar energy, semiconductor photocatalytic water splitting has attracted significant attention as a promising approach for green hydrogen production [4,5,6,7]. However, the current efficiency of photocatalytic hydrogen evolution remains insufficient to meet the demands of large-scale applications [8,9,10]. It is necessary to develop efficient and stable photocatalysts [11,12,13]. Recently, ZnIn_2_S_4_ (ZIS), a ternary metal chalcogenide with an asymmetric layered structure, has gained significant attention due to its excellent visible-light absorption ability and favorable physicochemical characteristics [14,15,16,17]. Nevertheless, the hydrogen evolution efficiency of pure ZIS is always restricted by intrinsic factors, such as low efficiency in photoinduced charge separation and transport, as well as the bulk susceptibility to hole oxidation during reactions, which compromises catalyst activity. Multiple strategies have been proposed to enhance the photocatalytic efficiency of ZIS, such as heterojunction construction [18], elemental doping [19,20,21], morphology design [22], cocatalyst deposition [23], and defect engineering [24]. Notably, constructing heterojunctions with staggered band alignments in semiconductors has proven to be an effective strategy for enhancing the separation efficiency of photoinduced charge carriers in catalytic systems [25,26]. Several methods for constructing heterojunction photocatalysts present promising pathways to improve the hydrogen evolution efficiency of ZIS-based systems [27,28,29], such as utilizing electrostatic interactions and van der Waals forces [30,31].

A strategically designed heterojunction photocatalyst enhances the light absorption in ZIS while preserving the reduction potential of photoinduced electrons. The heterojunction regulates the distribution of interfacial charges, effectively separating photoinduced charge carriers and facilitating the consumption of photoinduced holes. This prevents the self-oxidation of ZIS, thereby enhancing the stability of the catalyst. Consequently, the construction of heterojunction interfaces plays a critical role in optimizing the development of efficient composite photocatalysts [32,33]. Based on the migration pathways of photoinduced carriers, heterojunction photocatalysts are categorized into type I, type II, Z-scheme, and S-scheme structures [15]. Previous research has reported significant instances of heterojunction photocatalytic hydrogen evolution. For example, Li et al. [34] developed a novel Z-scheme heterojunction photocatalyst by anchoring ZIS nanosheets onto the surface of perovskite-structured CoTiO_3_. Compared with pristine ZIS, the CoTiO_3_/ZnIn_2_S_4_ photocatalyst exhibits enhanced photocatalytic hydrogen evolution activity, achieving a hydrogen production rate of 5.21 mmol g^−1^ h^−1^. This improvement is mainly attributed to the Z-scheme heterojunction, which promotes the efficient separation and migration of photoinduced carriers.

Among semiconductor catalysts, perovskite-structured SrTiO_3_ (STO) has garnered considerable attention for its non-toxicity and exceptional chemical stability [35]. The combination of this oxide with different substrates has made some remarkable achievements in photocatalytic hydrogen production; examples include the three-dimensional (3D) superstructure of g-C_3_N_4_ and reduced graphene oxide incorporated with Rh-doped SrTiO_3_ nanoparticles, forming ternary aerogels [36], as well as the ternary hierarchical SrTiO_3_/CdS/carbon nanosphere photocatalytic system [37]. However, the inherent limitations of pure STO, such as its wide bandgap, restrict its efficiency [38]. The formation of a robust heterojunction interface between ZIS and STO provides more active sites and facilitates interfacial charge transfer, and several STO-based heterojunction materials have been employed in diverse photocatalytic applications [39,40,41,42]. Considering the proper alignment of the energy bands between STO and ZIS, it is promising for hydrogen production applications. SrCrO_3_ is a type of strontium oxide with limited literature reports. Tamoor et al. [43] applied a SrCrO_3_/rGO nanohybrid as the supercapacitor electrode. Considering that SrCrO_3_ exhibits semiconductor behavior similar to STO [44,45], it can be regarded as a potential heterojunction photocatalyst. According to the best of our knowledge, the SrCrO_3_ has not been explored for the photocatalysts.

In the present article, we designed two efficient heterojunction photocatalysts for photocatalytic hydrogen evolution reactions under different single-wavelength light sources (λ = 400 and 420 nm). STO was synthesized using a hydrothermal method, with layered ZIS grown on its surface to form an STO@ZIS heterojunction. Meanwhile, SrCrO_3_ (SCO) was synthesized through a solid-state sintering method to construct the SCO@ZIS heterojunction. Experimental characterizations revealed that the optimized photocatalytic H_2_ evolution efficiency of STO@ZIS and SCO@ZIS reached 3.27 and 4.6 mmol g^−1^, respectively. The key factors contributing to the enhancement mechanism of photocatalysis were comprehensively evaluated, including the morphology and photoelectrochemical properties. These results indicate that both photocatalysts hold promise as heterojunctions for photocatalytic water splitting.

## 2. Materials and Methods

### 2.1. Preparation of Pristine ZnIn_2_S_4_

The synthesis of pure ZIS was conducted according to the procedure outlined by Tan et al. [46]. To synthesize 1 mmol of ZIS, 0.136 g of analytical-grade ZnCl_2_ (Shanghai, China) and 0.586 g of InCl_3_·4H_2_O (Shanghai, China) were dissolved in 30 mL ultrapure water with continuous stirring for 0.5 h. Then, 0.601 g of thioacetamide (TAA, Shanghai, China) was added to the solution, followed by another 0.5 h of stirring. The resulting mixture was transferred to a 100 mL Teflon-lined stainless-steel reaction autoclave and maintained at 160 °C for 2 h to facilitate the formation of ZIS. The product was washed three times by centrifugation, alternating between rinses with ultrapure water and ethanol. The product was dried at 60 °C for 12 h. The product was labeled as ZIS.

### 2.2. Preparation of SrTiO_3_

SrTiO_3_ was synthesized using the hydrothermal method. Analytical grade SrCl_2_·6H_2_O (Shanghai, China) was dissolved in 30 mL of ultrapure water and stirred for 0.5 h, followed by the dropwise addition of 3.4 mL of tetrabutyl titanate (Shanghai, China), with continued stirring for an additional 0.5 h. The suspension was transferred to an autoclave and reacted at 120 °C for 12 h. After cooling to room temperature, the product was washed three times by alternating centrifugation with ultrapure water and ethanol, subsequently drying at 60 °C for 12 h. The prepared sample was labeled as STO.

### 2.3. Preparation of SrCrO_3_

1 mmol of Sr(NO_3_)_2_ (Shanghai, China) and Cr_2_O_3_ (Shanghai, China) powders was placed in a mortar and ground for 0.5 h to ensure uniform mixing. The mixed powder was then placed in a tube furnace and calcined at 550 °C for 3 h. After removing the sample, it was ground for another 0.5 h and calcined again at 550 °C for an additional 3 h. The prepared sample was labeled as SCO.

### 2.4. Construction of the Heterojunction

A total of 21 mg of STO and SCO were each dispersed in 30 mL of deionized water and sonicated for 0.5 h. Subsequently, 0.136 g of ZnCl_2_ and 0.586 g of InCl_3_·4H_2_O were sequentially added to the solution, which was stirred for 0.5 h, followed by the addition of 0.601 g of TAA and further stirring for 0.5 h. The mixed solution was placed in an autoclave and reacted at 160 °C for 2 h. After cooling to room temperature, the product was washed three times by alternating centrifugation with ultrapure water and ethanol and subsequently dried at 60 °C for 12 h. The prepared samples were labeled as STO@ZIS and SCO@ZIS.

### 2.5. Characterization

X-ray diffraction (XRD) patterns were obtained using a Rigaku Ultima IV (Cu Kα target, operating at 40 kV and 40 mA). The morphologies and microstructure of the samples were observed using a ZEISS Sigma 360 scanning electron microscope (SEM), with an accelerating voltage ranging from 0.1 to 30 kV. Transmission electron microscopy (TEM), high-resolution TEM (HRTEM), and energy-dispersive X-ray (EDS) elemental mapping were performed using JEM-F200 (JEOL, Tokyo, Japan), with an accelerating voltage of 200 kV and magnifications ranging from 50 to 1.2 M. The Brunauer–Emmett–Teller (BET) specific surface area measurements were performed using ASAP 2460 (Micromeritics, Norcross, GA, USA). Pore size analysis was conducted using the Barrett–Joyner–Halenda (BJH) method.

The optical absorption spectra were measured using a UV–vis spectrophotometer (Hitachi UH4150, Tokyo, Japan). Analysis of the steady-state photoluminescence (PL) and time-resolved photoluminescence (TRPL) spectra was carried out using an Edinburgh FLS1000 spectrofluorometer. Electrochemical impedance and properties were characterized using a CHI760E electrochemical workstation (Shanghai, Chenhua Instrument Co. Ltd., Shanghai, China). A photocatalyst (5 mg) was uniformly dispersed in 1 mL of an ethanol solution containing 10 vol% Nafion, which was then coated onto a 1 cm × 1 cm FTO substrate as the working electrode. A Pt sheet and an Ag/AgCl electrode served as the counter and reference electrodes, respectively, with a 0.5 M Na_2_SO_4_ solution as the electrolyte. The electrochemical impedance spectroscopy (EIS) was conducted in a 0.5 M Na_2_SO_4_ solution under a frequency range between 0.1 Hz and 100 kHz. The amplitude of applied sine wave potential in each case was 10 mV. The linear scan voltammetry (LSV) curves and chopped linear sweep voltammograms were performed in a three-electrode system with a scan range from 0 to −1 V at a scan rate of 10 mV/s. The chopped linear sweep voltammogram switch indicator interval was 10 s. Photocurrent experiments were carried out under a 300 W Xe lamp, with testing performed at a potential of 0.5 V. Both EIS and LSV measurements were conducted under light exposure. In the photocurrent test, the system was cycled as follows: the light was turned off for the first 0–20 s, followed by 20 s of light exposure, then 20 s of the light off, repeating this cycle six times for a total duration of 265 s, with testing conducted at 0.5 V. The Mott–Schottky measurements were performed over a voltage range of ±1 V, with a frequency of 1200 Hz.

Photocatalytic hydrogen production tests were performed in a sealed vacuum quartz container (50 mL), with 5 mg of the catalyst ultrasonically dispersed in 20 mL of an aqueous solution containing 20 vol% triethanolamine (TEOA). A 300 W Xe lamp (λ = 400 and 420 nm) served as the light source, and the generated gas was analyzed via gas chromatography. The illumination area is 28.68 cm^2^. The average optical power density measured by the five-point method is 93.81 and 85.113 mW/cm^2^ at the light intensity 400 and 420 nm, respectively. The reactor was evacuated and filled with N_2_ before irradiation at room temperature. The molar hydrogen evolution per unit mass of the photocatalyst over time was calculated as the performance indicator for photocatalytic activity.

## 3. Results and Discussion

### 3.1. Morphology and Structure

**Figure 1:**
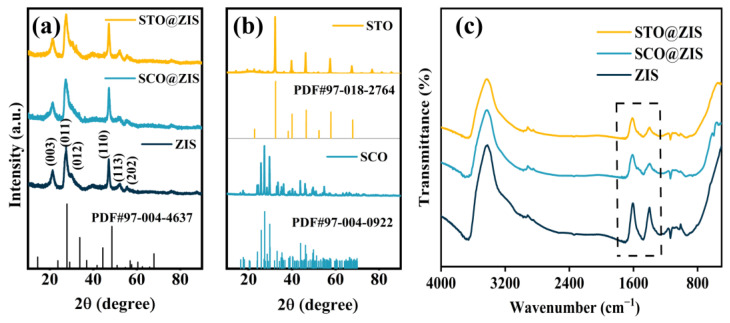
(**a**) XRD patterns of STO@ZIS, SCO@ZIS, and ZIS, (**b**) XRD patterns of STO and SCO, and (**c**) FTIR patterns of STO@ZIS, SCO@ZIS, and ZIS.

STO@ZIS and SCO@ZIS heterojunction photocatalysts were prepared using a hydrothermal method, and the crystal structures of the synthesized samples were characterized by XRD. As shown in Figure 1a, the diffraction peaks of the as-prepared ZIS could be indexed to the standard PDF#97-004-4637 (a = b = 3.85 Å, c = 12.34 Å), confirming that the synthesized ZIS belongs to the hexagonal system. Characteristic peaks at 21.59°, 27.69°, 30.45°, 47.18°, 52.44°, and 57.12° correspond to the crystal planes of (003), (011), (012), (110), (113), and (202), respectively [47]. Figure 1b shows that the XRD patterns of the prepared oxide substrate exhibit sharp diffraction peaks. The diffraction peaks of pure STO and SCO could be indexed to the tetragonal crystal system (PDF#97-018-2764, a = b = 5.51 Å, c = 7.81 Å) and the monoclinic crystal system (PDF#97-004-0922, a = 7.07 Å, b = 7.38 Å, c = 6.74 Å), respectively. Notably, the XRD patterns of the heterojunction catalysts retain the characteristic peaks of ZIS, along with the principal characteristic peaks of STO and SCO. To further examine the characteristic functional groups of prepared photocatalysts, Fourier transform infrared spectroscopy (FTIR) analysis was conducted. As depicted in Figure 1c dotted boxes, all photocatalysts exhibit similar characteristic absorption peaks at 1600 and 1400 cm^−1^, which correspond to hydroxyl functional groups and surface-adsorbed water molecules, respectively [48]. The combined results from the XRD and FTIR analyses confirmed the synthesis of STO@ZIS and SCO@ZIS heterojunction catalysts. Furthermore, these findings demonstrate that the incorporation of ZIS did not induce alterations in the phase structure of the substrate oxides.

**Figure 2:**
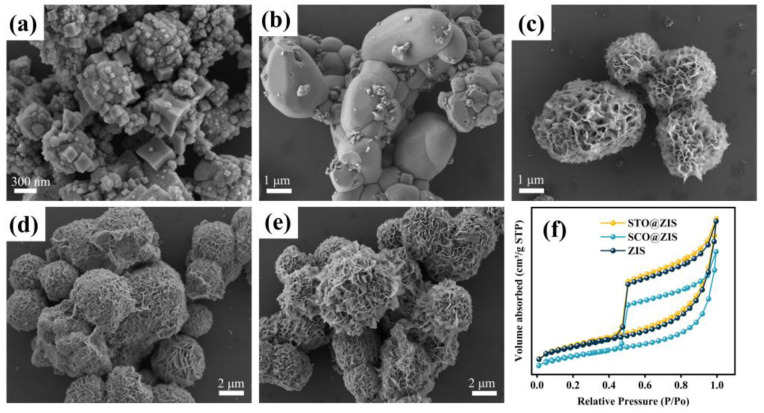
SEM images of (**a**) STO, (**b**) SCO, (**c**) ZIS, (**d**) STO@ZIS, (**e**) SCO@ZIS, and (**f**) N_2_ adsorption–desorption isotherms.

Figure 2 presents the surface morphologies of the prepared samples. As depicted in Figure 2a, the prepared STO consists of numerous stacked nanocubes. Figure 2b shows that SCO is composed of aggregated irregular nanospheres. As shown in Figure 2c, pristine ZIS displays a nanoflower-like microsphere structure formed from stacked nanosheets, with approximately 4–10 μm diameters. Figure 2d,e shows the morphologies of heterojunction photocatalysts, where STO@ZIS and SCO@ZIS are characterized by vertically aligned ZIS nanosheets on the surface of STO and SCO, forming thin nanosheets.

The specific surface area and pore structure of prepared samples were determined through N_2_ adsorption–desorption isotherms. As shown in Figure 2f, the specific surface areas of STO, SCO, and sheet-like ZIS are 10.00, 0.64, and 77.26 m^2^ g^−1^, with average pore diameters of 12.38, 23.56, and 5.16 nm, respectively. All the prepared samples exhibit mesoporous characteristics. The specific surface areas of the heterojunction catalysts STO@ZIS and SCO@ZIS are 81.16 and 53.43 m^2^ g^−1^, respectively, with average pore diameters of 5.07 and 5.14 nm. A comparison of the results indicates a significant increase in the specific surface area of the heterojunction catalysts. Concurrently, the average pore size decreases due to the coverage of voids by the growth of ZIS on the oxide substrates, resulting in the average pore size similar to that of pure ZIS. The textural characteristics of the heterojunction catalysts create advantageous conditions for improving photocatalytic activity.

**Figure 3:**
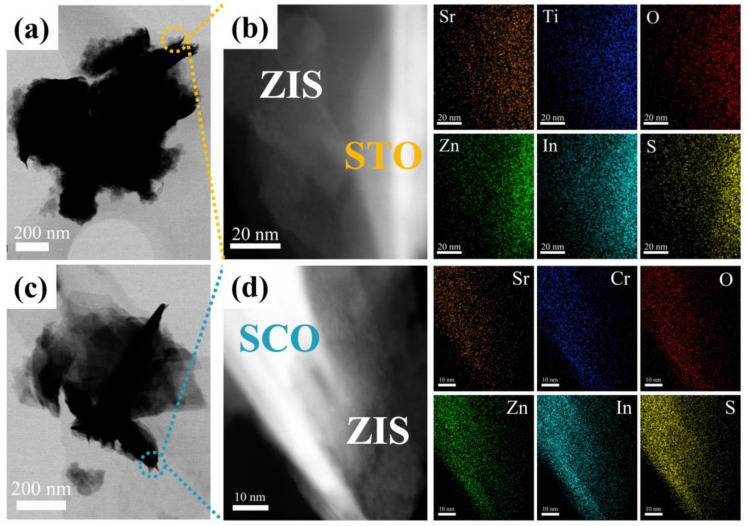
TEM of (**a**) STO@ZIS, (**c**) SCO@ZIS, EDS mapping of (**b**) STO@ZIS, and (**d**) SCO@ZIS.

As shown in Figure 3, TEM confirms that the heterojunction catalysts STO@ZIS and SCO@ZIS are structured with ZIS nanosheets coating the surfaces of the oxide substrates. Specifically, EDS mapping of STO@ZIS and SCO@ZIS demonstrates a consistent distribution of Zn, In, and S elements within the samples, indicating a uniform growth of ZIS nanosheets on the surfaces of the oxide substrates. The presence of Sr, Ti, O, and Cr elements, which constitute the oxide substrates, is clearly visible, consistent with the composite structure. Furthermore, the distinct boundary observed between the oxide substrates and ZIS suggests tight interfacial contact, which facilitates the efficient transport of photoinduced charges and improves their separation and transfer efficiency.

### 3.2. Optical Properties and Band Structure

**Figure 4:**
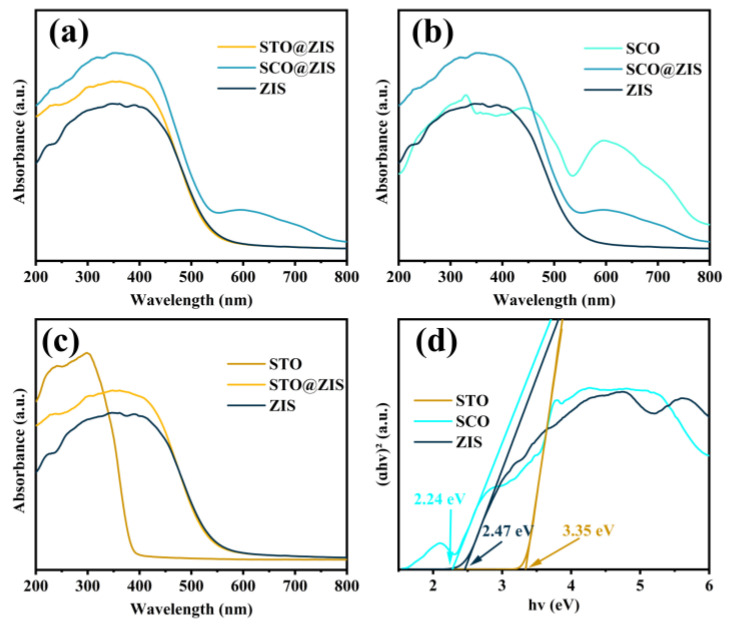
UV–vis DRS spectra of (**a**) heterojunction photocatalysts, (**b**,**c**) oxide substrates, and (**d**) Tauc plot.

As shown in Figure 4, the optical features of samples were characterized using the UV–vis diffuse reflectance spectroscopy (UV–vis DRS). Figure 4a shows that STO@ZIS and SCO@ZIS display higher light response intensities, with SCO@ZIS exhibiting a redshift. These characteristics are advantageous for improving the catalytic activity of the heterojunction catalysts. Figure 4b,c illustrates the light absorption capacity of the heterojunction catalyst in comparison to the oxide substrates, demonstrating that the heterojunction catalyst displays higher light response intensities. With the growth of ZIS on the surfaces of the oxide substrates, the optical absorption properties of the heterojunction catalysts become comparable to those of ZIS, with enhanced absorption in the visible light range. The absorption edges of STO, SCO, and pristine ZIS are approximately 380, 530, and 520 nm, respectively. The band gap values of the oxide substrates and pristine ZIS were obtained based on ${\left(\partial hv\right)}^{1/n}=A\left(hv-E_{g}\right)$. As shown in Figure 4d, the calculated bandgap of STO, SCO, and pristine ZIS is 3.35 eV, 2.24 eV, and 2.47 eV, respectively.

**Figure 5:**
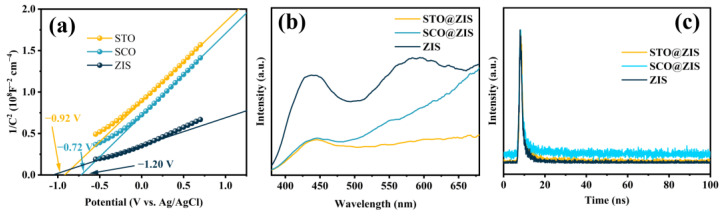
(**a**) Mott–Schottky plots, (**b**) PL spectra, and (**c**) TRPL spectra.

The energy band structure was characterized using Mott–Schottky (M-S) curves at an AC frequency of 1200 Hz. As shown in Figure 5a, all prepared samples are identified as n-type semiconductors, determined by the positive slope of the tangent line to the M-S curves. Furthermore, the flat band potential (*E*_fb_) of STO, SCO, and ZIS was determined to be −0.92 V, −0.72 V, and −1.2 V vs. Ag/AgCl, respectively. As is known, the conduction band positions (*E*_C_) of n-type semiconductors are approximately 0.2 V more negative than *E*_fb_ [49,50]. Accordingly, the *E*_C_ for STO, SCO, and ZIS were estimated to be −1.12 V, −0.92 V, and −1.4 V, respectively [51,52]. Based on the relative relationship between the valence band (*E*_V_), *E*_C_, and *E*_g_ (*E*_g_ = *E*_C_ − *E*_V_), the valence band positions of STO, SCO, and ZIS were estimated to be 2.23 V, 1.32 V, and 1.07 V, respectively. These values can be converted to 2.43 V, 1.52 V, and 1.27 V vs. NHE.

To evaluate the influence of heterojunction photocatalysts on the separation and transfer of photoinduced charges, PL and TRPL spectra were employed to characterize the photoinduced charge recombination of prepared photocatalysts under 314 nm excitation. As shown in Figure 5b, pristine ZIS exhibits a strong emission signal, indicating the significant recombination of photoinduced electron-hole pairs, which suppresses photocatalytic activity. However, after decorating the oxide substrate surfaces with ZIS, the PL peak intensities of the heterojunction catalysts STO@ZIS and SCO@ZIS decreased significantly, indicating that the formed heterojunction effectively suppresses photoinduced carrier recombination. Meanwhile, the TRPL spectra provide insights into charge carrier migration dynamics, as shown in Figure 5c. The average fluorescence lifetime of pristine ZIS is 2.28 ns, shorter than that of STO@ZIS (3.56 ns) and SCO@ZIS (4.80 ns), confirming that the heterojunction interface provides a rapid channel for photoinduced charge transfer. Additionally, long-lived photoinduced electrons have more opportunities to participate in photocatalytic hydrogen evolution. Overall, the heterojunction photocatalysts STO@ZIS and SCO@ZIS exhibit stronger light absorption, faster charge migration rates, and more effective charge separation capabilities compared to pristine ZIS.

### 3.3. Photoelectrochemical and Photocatalytic Performance

**Figure 6:**
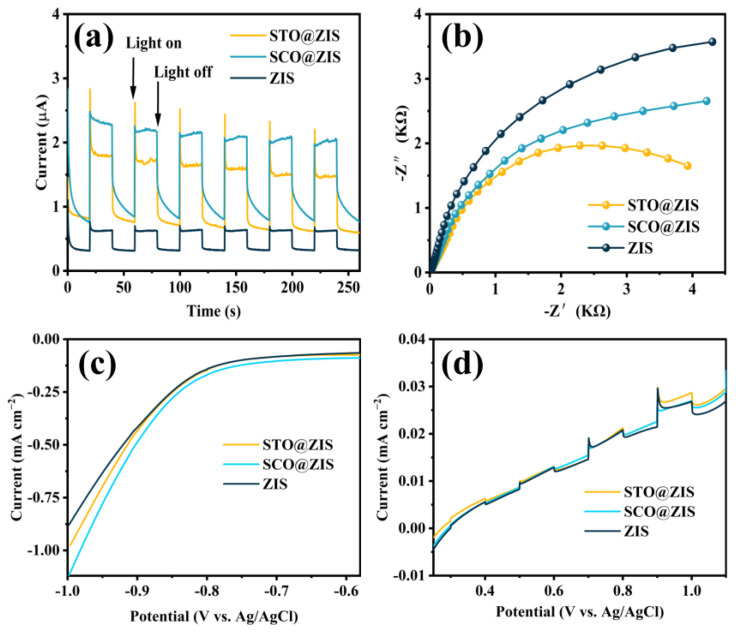
(**a**) Photocurrent responses, (**b**) EIS plots, (**c**) LSV curves, and (**d**) chopped linear sweep voltammetry.

To further evaluate the positive effects of heterojunction construction on catalytic performance, transient photocurrent response, EIS, LSV, and chopped linear sweep voltammetry were conducted to investigate the interfacial charge transfer behavior of the heterojunction catalysts. As shown in Figure 6a, all samples demonstrated rapid response during visible light on/off cycles. Regarding comprehensive optical properties, the heterojunction catalysts display stronger photocurrent response signals than pristine ZIS, indicating that the enhanced light-harvesting capacity allows the heterojunction catalysts to generate more photoinduced charge carriers. Specifically, STO@ZIS generates a higher instantaneous current at the moment of illumination, with electron transport efficiency stabilizing over continued exposure. SCO@ZIS shows a higher average current density, consistent with the fluorescence lifetime results. In Figure 6b, the interfacial charge transfer behavior of prepared samples is presented, as analyzed through EIS. The results showed that the heterojunction catalysts exhibit smaller radii in the Nyquist plots, indicating reduced electrochemical impedance. This confirms that the heterojunction construction reduces interfacial charge migration resistance, thereby enabling the more efficient participation of charges in catalytic reactions. As shown in Figure 6c, SCO@ZIS requires smaller applied voltages than STO@ZIS and pristine ZIS at the same current. It shows that a smaller driving force can stimulate the electrons in the heterojunction catalysts to participate in the photocatalytic reaction. Figure 6d shows the different photocurrent densities of the prepared samples under the conditions of interspaced illumination.

**Figure 7:**
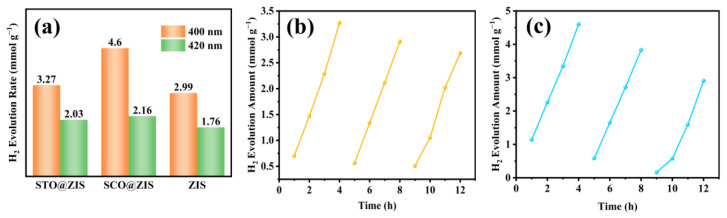
(**a**) Hydrogen evolution efficiency of the photocatalyst, and (**b**,**c**) the stability test of STO@ZIS and SCO@ZIS in three cycles.

The photocatalytic hydrogen evolution performance of prepared heterojunction catalysts was investigated without the additional cocatalysts. As depicted in Figure 7a, pristine ZIS exhibited relatively low hydrogen evolution efficiency of 2.99 and 1.76 mmol g^−1^ under 400 and 420 nm, which can be attributed to the rapid recombination of photoinduced electron-hole pairs. After ZIS was grown on the surface of the oxide substrate, the hydrogen evolution efficiency of STO@ZIS and SCO@ZIS increases to 3.27 and 4.6 mmol g^−1^, under a 400 nm light source. The hydrogen evolution efficiency of STO@ZIS and SCO@ZIS reaches 2.03 and 2.16 mmol g^−1^, under a 420 nm light source. Among these, SCO@ZIS exhibited the optimal hydrogen evolution efficiency of 4.6 mmol g^−1^, which is 1.54 times that of pristine ZIS (2.99 mmol g^−1^). As displayed in Figure 7b,c, the hydrogen evolution of STO@ZIS and SCO@ZIS exhibited no significant decrease after three cycles.

**Figure 8:**
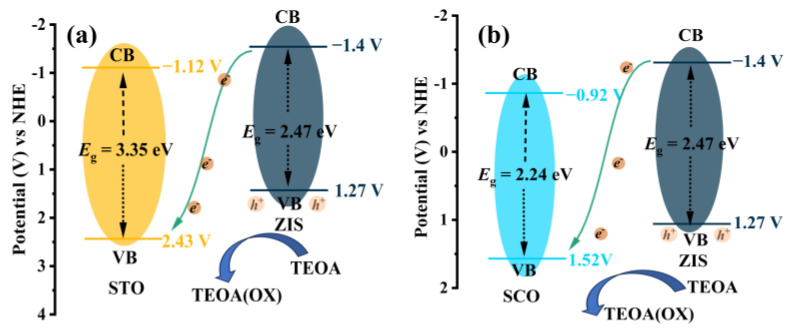
Charge transfer mechanism of (**a**) STO@ZIS, (**b**) SCO@ZIS.

Considering the band structures of STO, SCO, and ZIS, a potential mechanism for photocatalytic hydrogen evolution can be inferred. As illustrated in Figure 8a, STO@ZIS promotes the transfer of photogenerated carriers from the ZIS side to the STO side due to the ultraviolet absorption of STO. Subsequently, these carriers participate in the surface hydrogen evolution reaction. The photogenerated holes of ZIS are consumed by the sacrificial agent, forming a typical type-II heterojunction that promotes the spatial separation of photogenerated carriers, enhancing the hydrogen evolution efficiency of the photocatalyst. A similar migration path for photoinduced charge carriers is observed in SCO@ZIS, as illustrated in Figure 8b.

## 4. Conclusions

This study highlights the successful design of novel SrTiO_3_@ZnIn_2_S_4_ and SrCrO_3_@ZnIn_2_S_4_ heterojunctions for enhanced photocatalytic hydrogen evolution. The synergy between ZnIn_2_S_4_ and the oxide substrates not only promotes efficient light harvesting and charge separation but also reduces interfacial charge transfer resistance. The observed increase in hydrogen evolution efficiency, with enhancements of 1.09 and 1.54 times compared to pristine ZnIn_2_S_4_, underscores the importance of optimizing heterojunctions for photocatalytic applications. This work paves the way for further exploration of heterojunction designs with tailored interfacial properties to achieve higher photocatalytic performance. Future studies could focus on incorporating cocatalysts, exploring different oxide materials, and optimizing the interfacial contact for even more efficient water splitting systems.

## Data Availability

The dataset is available upon request from the authors.
